# Novel Dual-Coenzyme Specificity and Thermostability of Malate Dehydrogenase Identified in the Cyanobacterium *Microcystis aeruginosa* PCC7806

**DOI:** 10.3390/ijms262110313

**Published:** 2025-10-23

**Authors:** Yadong Ge, Yifan Wu, Bo Zhou, Xiaojie Wu, Yu Ren, Jialin Zhu, Yali Ge

**Affiliations:** 1 Anhui Provincial Key Laboratory of Molecular Enzymology and Mechanism of Major Metabolic Diseases, College of Life Sciences, Anhui Normal University, Wuhu 241002, China; geyd@ahnu.edu.cn (Y.G.); wuyf1226@ahnu.edu.cn (Y.W.); 2 Auhui Provincial Engineering Research Centre for Molecular Detection and Diagnostics, College of Life Sciences, Anhui Normal University, Wuhu 241002, China; 3 School of Ecology and Environment, Anhui Normal University, Wuhu 241002, China; 2421012826@ahnu.edu.cn (B.Z.); 2421012801@ahnu.edu.cn (X.W.); 2321012758@ahnu.edu.cn (Y.R.); 2321012764@ahnu.edu.cn (J.Z.)

**Keywords:** cyanobacteria, *Microcystis aeruginosa* PCC7806, malate dehydrogenase, enzymatic characterization, dual coenzyme specificity, thermostability, kinetics, molecular evolution

## Abstract

Malate dehydrogenase (MDH) is a key energy metabolic enzyme with distinct coenzyme specificity for either NAD^+^ or NADP^+^ in all domains of life. Here, we characterize a novel MDH from the bloom-forming cyanobacterium *Microcystis aeruginosa* PCC7806 (MaMDH), which displays dual-coenzyme specificity with comparable efficiency for both NAD^+^ and NADP^+^, albeit with a slight preference for NAD^+^. MaMDH exists as a 72.1 kDa homodimer with a subunit mass of 36.2 kDa in solution. Kinetic measurements yielded *K*_m_ values of 33.140 μM for NAD^+^ and 113.200 μM for NADP^+^, with a *k*_cat_ ratio (NAD⁺/NADP⁺) of 3.64. The enzyme exhibited optimal activity at pH 8.0 and 40 °C, along with notable thermostability, retaining over 90% activity after incubation at 70 °C for 20 min. Through structure-guided mutagenesis of the predicted coenzyme-binding motif, we shifted MaMDH cofactor preference from NAD^+^ toward NADP^+^, supporting the hypothesis that dual-specificity MDHs may represent evolutionary intermediates in the emergence of NADP^+^-dependent chloroplast MDHs. This study provides new insights into the molecular evolution mechanisms of coenzyme specificity within the MDH family.

## 1. Introduction

Malate dehydrogenase (MDH) is a ubiquitous enzyme that plays an essential role in cellular respiration throughout all domains of life. It catalyzes the reversible conversion of oxaloacetate to L-malate, concomitant with the oxidation or reduction of the nicotinamide cofactors NAD(H) or NADP(H) [1,2,3]. As a pivotal regulatory enzyme in the tricarboxylic acid (TCA) cycle, MDH is categorized according to cofactor specificity into NAD-dependent MDH (NAD-MDH) (EC 1.1.1.37) and NADP-dependent MDH (NADP-MDH) (EC 1.1.1.82) forms [4]. The majority of bacterial and archaeal MDHs utilize NAD^+^, while eukaryotic isoforms—comprising cytosolic (cMDH), mitochondrial (mMDH), glyoxysomal (gMDH), and chloroplastic NAD-dependent MDH (cnMDH)—are also predominantly NAD⁺-dependent, with the exception of chloroplastic NADP-MDH (chMDH) [2,5]. Notably, MDH from the archaeon *Metallosphaera sedula* (MsMDH) has been reported to exhibit dual cofactor specificity, utilizing both NADH and NADPH [6].

The MDH family belongs to the 2-ketoacid NAD(P)^+^-dependent dehydrogenases superfamily [2,3], and can be phylogenetically divided into three subfamilies: cluster I, cluster II, and cluster III [3,7]. Cluster I includes NAD-MDHs from certain eubacteria (including the cyanobacterium *Synechocystis*), archaeal LDH-like MDHs, as well as lactate dehydrogenases (LDHs) from archaea, eubacteria, animals, and plants, representing the most ancient branch of the MDH evolutionary tree. Cluster II MDHs comprises NAD-MDHs from eukaryotic mitochondria, peroxisomes, and glyoxysomes, as well as plastid-localized in eukaryotes and yeast. Cluster III MDHs includes cytosolic NAD-MDHs from animals, plants, and eubacteria, along with chloroplastic NADP-MDHs from plants [7].

NAD-MDH participates in various intracellular metabolic pathways, including amino acid biosynthesis, the malate–aspartate shuttle, gluconeogenesis, and lipogenesis, playing a crucial role in aerobic energy production within organisms [4,8,9]. NADP-MDH is primarily involved in the photosynthetic dicarboxylic acid cycle in plant chloroplasts and helps maintain redox balance between the chloroplast and cytosol [10,11]. It has also been extensively studied for its role as a malate valve in plants [11,12,13]. Recent evidence suggests that MDH contributes to metabolic plasticity in tumor cells and catalyzes the formation of an oncometabolite, thereby promoting tumorigenesis [14,15].

Due to its widespread phylogenetic distribution, MDH serves as a valuable model for investigating molecular evolution and cellular complexity [16]. Notably, NAD-MDH represents the predominant form within the enzyme family. Phylogenetic evidence further indicates that plant chloroplastic NADP-MDH evolved from cytosolic NAD-MDH precursors, rather than from the gene encoding the plastid NAD^+^-specific isoenzyme [7]. Site-directed mutagenesis studies aimed at modifying coenzyme specificity had simulated this evolutionary mechanism, and to a certain extent, validated the above-mentioned hypothesis of MDH family system evolution. Therefore, dual-coenzyme-dependent MDH, representing a transitional form in the evolutionary shift of coenzyme specificity in MDH, is of significant phylogenetic importance.

Despite extensive studies on malate dehydrogenases (MDHs), the evolution of NADP-dependent forms from their NAD-dependent ancestors remains unclear. Dual-coenzyme-specific MDHs, proposed as potential evolutionary intermediates, are exceptionally rare and poorly characterized. To date, only the MDH from *Metallosphaera sedula* (MsMDH) has been described; however, its phylogenetic position and kinetic properties do not fully support a transitional role toward NADP⁺ specificity. This poses an open question worthy of in-depth study: can a natural dual-coenzyme MDH from cyanobacteria—the putative ancestors of plant chloroplasts—function as a true evolutionary link between NAD- and NADP-dependent MDHs? To enhance our understanding of this question, we characterized the MDH from *Microcystis aeruginosa* PCC7806 (MaMDH), a ubiquitous, bloom-forming cyanobacterium known for producing microcystins and causing harmful algal blooms [17]. Our work provides the first biochemical and evolutionary analysis of a cyanobacterial dual cofactor specificity MDH, directly testing the hypothesis that such enzymes represent transitional forms in the evolution of chloroplast NADP-MDHs.

## 2. Results

### 2.1. Bioinformatics Analysis

**Figure 1:**
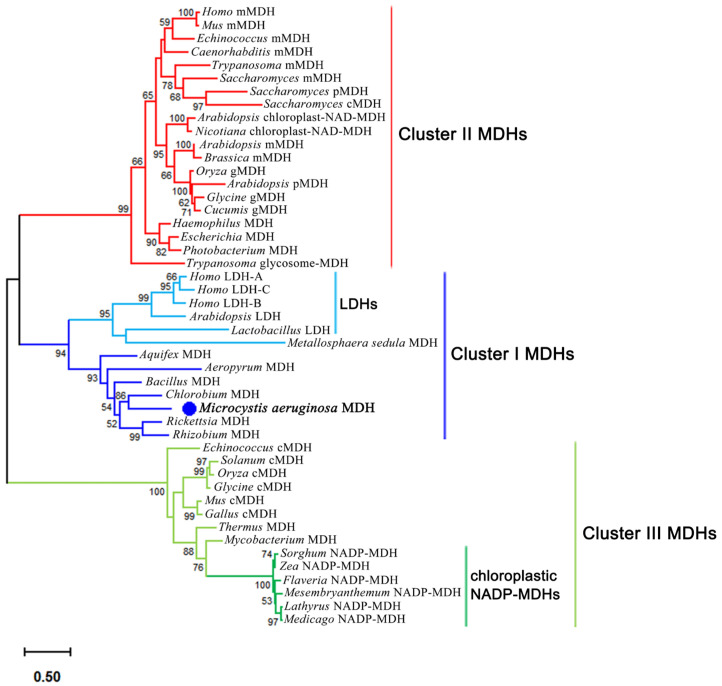
Protein maximum likelihood tree of MDH family. The phylogenetic tree was constructed by MEGA 12.0 with 1000 bootstrap replicates. cMDH: cytosolic MDH; mMDH: mitochondrial MDH; gMDH: glyoxysomal MDH; pMDH:peroxisomal. The MaMDH was targeted by blue dot.

The evolutionary history of the MDH family was reconstructed through phylogenetic analysis (Figure 1), which positioned MaMDH within the ancestral cluster I subfamily. MaMDH exhibited a closer phylogenetic affinity to prokaryotic and archaeal NAD-MDHs, clearly distinguishing it from the dual-coenzyme-dependent MsMDH. MsMDH is clustered closely with the branches representing LDHs, indicating that it belongs to the LDH-like MDH subgroup. This implies that MaMDH is a novel dual-coenzyme-dependent MDH.

**Figure 2:**
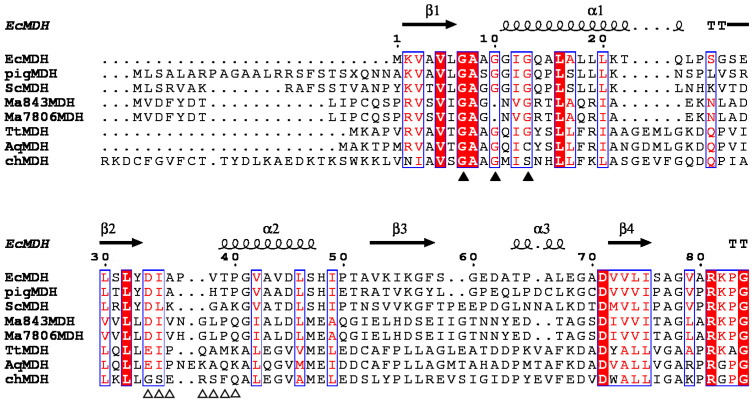
Structure-based amino acid sequence alignment. ▲: Malate dehydrogenase coenzyme binding conserved site; △: NADPH binding site of malate dehydrogenase in chloroplast. EcMDH: *Escherichia coli* MDH (Accession: AAN82431.1); ScMDH: Saccharomyces cerevisiae S288C MDH (Accession: NP_012838.1); chMDH: *Sorghum vulgare* MDH (Accession: M31965.1); TtMDH: *Thermus thermophilus* MDH (Accession: 4KDE_A); AqMDH: *Aquaspirillum arcticum* MDH (Accession: Q9ZF99.3); Ma843MDH: *Microcystis aeruginosa* NIES-843 MDH (Accession: WP_012264466.1); Ma7806MDH: *Microcystis aeruginosa* PCC7806 MDH (Accession: WP_002747035.1); pigMDH: *Sus scrofa* MDH (Accession: P00346.2). The structure of EcMDH (PDB ID: 6KA1) was downloaded from the PDB database. The figure was created by ESPript 3.0.

The N-terminal consensus sequence of MDHs is involved in the binding of the ADP portion of NAD^+^ and is considered a characteristic signature [18,19]. Structure-based sequence alignment showed that the MaMDH contains a conserved N-terminal sequence (G^20^AGNVG^25^) similar to other MDHs from bacteria, archaeons, animals, and plants (Figure 2). Alignment with NAD-MDHs and NADP-MDHs identified seven residues (Asp44, Ile45, Val46, His47, Gly48, Leu49, and Pro50) as putative coenzyme-binding sites in MaMDH (Figure 2).

### 2.2. Expression and Purification of Recombinant MaMDH

**Figure 3:**
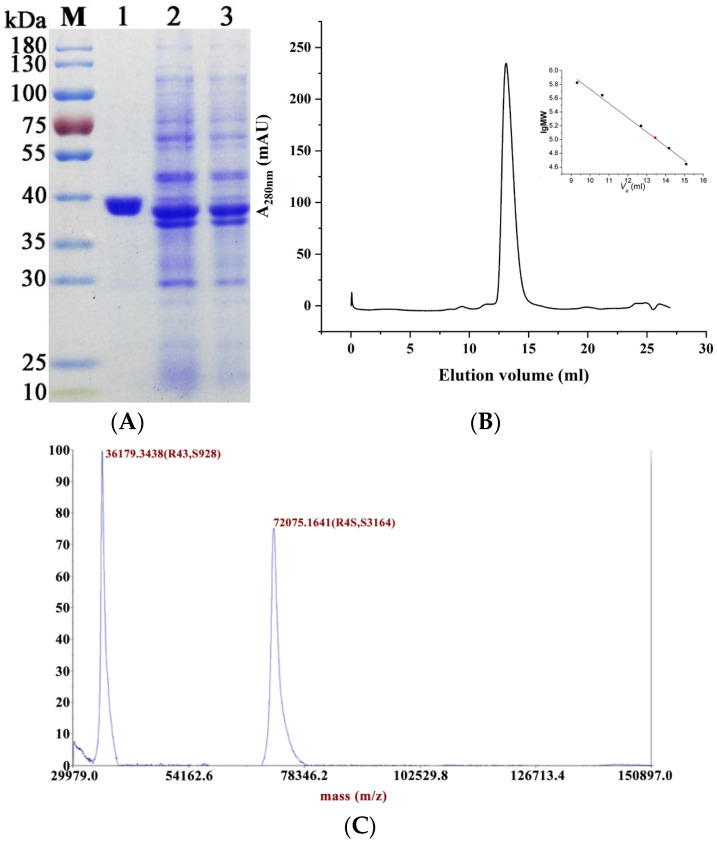
Overexpression and purification of MaMDH. (**A**) SDS-PAGE analysis. M: protein molecular mass markers; lane 1: purified MaMDH; lane 2: supernatant of crude extract; lane 3: precipitation of crude extract. (**B**) Gel filtration chromatography analysis. The standard curve shows the logarithmic molecular weight against elution volume; *V*_e_ of the MaMDH was 13.39 mL; MaMDH was represented by the red circle. (**C**) MALDI-TOF-MS analysis. Single and double-charged molecular ions were observed at 36,179.3438 and 72,075.1641 Da per electron unit, respectively. BSA was used as the external standard.

MaMDH consists of 325 amino acids with a predicted molecular mass of 35.0 kDa. SDS-PAGE showed a distinct band at 35–40 kDa (Figure 3A). Gel filtration chromatography showed that the molecular weight of the target protein was approximately 77.8 kDa in solution (Figure 3B). MALDI-TOF-MS analysis revealed two peaks at 36,179.3438 and 72,075.1641 Da (Figure 3C), indicating that recombinant MaMDH forms homodimers (72.1 kDa), with each subunit weighing 36.2 kDa.

### 2.3. Effects of pH, Temperature and Metal Ions on MaMDH Activity

**Figure 4:**
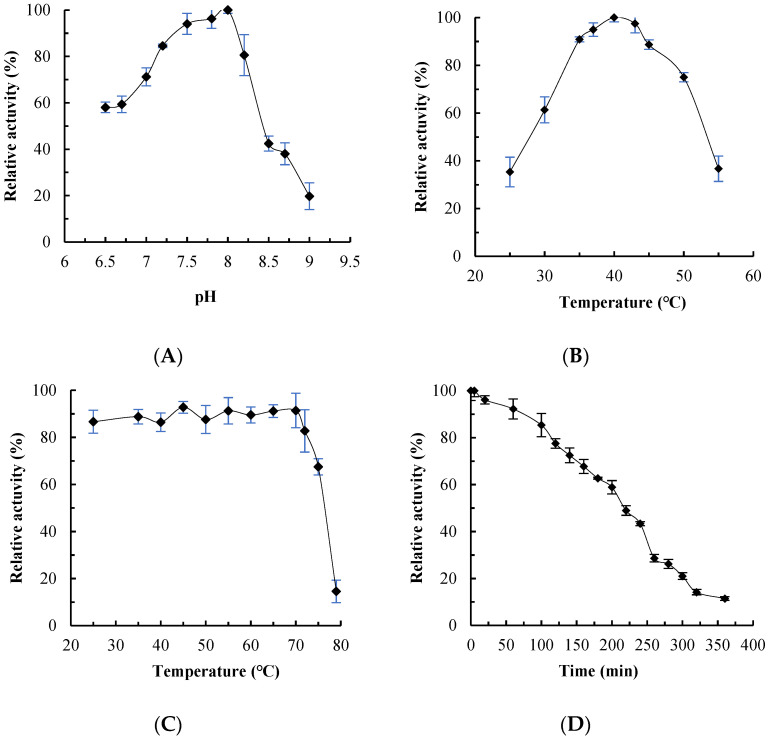
(**A**) Effects of pH on the activity of MaMDH; (**B**) Effects of temperature on the activity of MaMDH; (**C**) Effects of temperature on the stability of MaMDH; (**D**) The thermal inactivation profiles of MaMDH at 55 °C.

The effects of pH and temperature on MaMDH activity were assessed using NADH as the coenzyme. Enzyme activity increased initially with rising temperature and pH, then decreased sharply. Optimal activity was observed at pH 8.0 (Figure 4A) and 40 °C (Figure 4B). After incubation at 70 °C or lower for 20 min, MaMDH retained approximately 90% of its original activity. At 75 °C, it lost about 30% of its activity, and was almost completely inactivated at 80 °C (Figure 4C). Thermostability at 55 °C was also evaluated: MaMDH retained over 90% of its initial activity after 60 min and more than 70% after 140 min, with a half-life (*t*_1/2_) of approximately 220 min (Figure 4D).

Although MDHs typically do not require metal ions for catalysis, the addition of Na^+^, Li^+^, or Rb^+^ slightly stimulated MaMDH activity. Treatment with 2 mM Mn^2+^, Ca^2+^, or Mg^2+^ enhanced MaMDH activity by 28.1%, 30.8%, and 35.8%, respectively. In contrast, 2 mM Zn^2+^ nearly completely inactivated the enzyme. Activity decreased by over 80% in the presence of 2 mM Co^2+^ or Ni^2+^, and by about 20% with 2 mM Cu^2+^. K^+^ had no effect on MaMDH activity (Table 1). MaMDH activity decreased with increasing concentrations of dimethyl sulfoxide (DMSO) and Triton X-100. The activity was reduced by approximately 30% and 50% in the presence of 8% DMSO and 8% Triton X-100, respectively (Table 1).

### 2.4. Kinetics Analysis

In vitro, MaMDH primarily catalyzes the reduction of oxaloacetate (OAA), with negligible activity in the oxidative direction; thus, only reductive reaction data are presented. Kinetic analyses using NADH and NADPH as cofactors revealed that recombinant MaMDH exhibits similar affinity and catalytic efficiency for both coenzymes, indicating its dual-coenzyme dependency, albeit with a slight preference for NADH. The *K*_m_ values for NAD^+^ and NADP^+^ were 33.140 μM and 113.200 μM, respectively, while the *k*_cat_ value for NAD^+^ (31.688 s^−1^) was only 3.64-fold higher than that for NADP^+^ (8.710 s^−1^) (Table 2).

**Figure 5:**
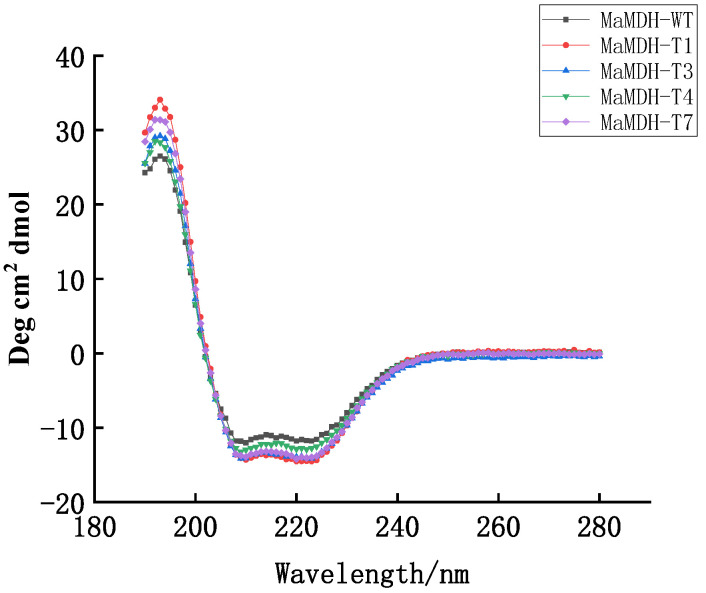
Circular dichroism spectra of the wild-type MaMDH and its mutants.

Based on the known NADP^+^-binding site of *Thermus flavus* MDH (chMDH), the NAD^+^-binding site (D^44^IVHGLP^50^) in MaMDH was replaced with the NADP^+^-binding sequence (G^148^SERSFQ^154^) from chMDH [20,21]. Four mutants were constructed: MaMDH-T1 (D^44^G); MaMDH-T3 (D^44^G/I^45^S/G^48^S); MaMDH-T4 (D^44^G/I^45^S/H^47^R/G^48^S); and MaMDH-T7 (D^44^G/I^45^S/V^46^E/H^47^R/G^48^S/L^49^F/P^50^Q). Circular dichroism spectra confirmed that all mutants retained their secondary structures after mutagenesis (Figure 5).

The *K*_m_ values for NAD^+^ (*K*_m_^NADH^) and NADP^+^ (*K*_m_^NADPH^) of the four mutants are listed in Table 2. MaMDH-T4 showed a 6.69-fold higher preference for NADP^+^ over NAD^+^, with its coenzyme specificity ratio (NAD^+^/NADP^+^) decreasing from 12.427 to 0.149 (an 83.15-fold change). Mutants MaMDH-T3 and MaMDH-T7 also exhibited significant changes in catalytic efficiency (*k*_cat_/*K*_m_) when switching from NAD^+^ to NADP^+^, with alterations of 63.01-fold and 46.16-fold, respectively. These results indicate that, compared to wild-type MaMDH, mutants T3, T4, and T7 retained dual-coenzyme utilization capacity but showed a pronounced shift from NAD^+^-predominant affinity to NADP^+^ preference. mutant MaMDH-T1 showed only a modest increase (1.54-fold) in catalytic efficiency toward NADP^+^.

## 3. Discussion

Malate dehydrogenase (MDH) is a central enzyme in aerobic respiration, playing a critical role in energy metabolism across the majority of life on Earth [16]. Its evolutionary conservation and ubiquity make it an excellent model for studying the history of life and the emergence of aerobic respiration and eukaryogenesis. In this study, the cluster I homodimeric MaMDH was characterized. Phylogenetic analysis (Figure 1) reveals that although MaMDH groups with prokaryotic and archaeal NAD-dependent MDHs, it is phylogenetically distinct from the dual-coenzyme-dependent archaeal MsMDH, which clusters within the LDH-like MDH subgroup along LDHs branches. Kinetic parameter assessments further confirmed that MaMDH represents a novel dual-coenzyme-dependent MDH.

MDH is typically a multimeric enzyme, often forming dimers or tetramers [2,8]. SDS-PAGE indicated a subunit molecular mass of 35–40 kDa for recombinant MaMDH (Figure 3A), and gel filtration chromatography revealed that MaMDH in solution eluted as a single symmetric peak with a molecular mass of approximately 77.8 kDa (Figure 3B), indicating that MaMDH exists as a homodimer. MALDI-TOF-MS confirmed its dimeric state in solution, with a total mass of 72.1 kDa and subunits of 36.2 kDa each (Figure 3C). Thus, MaMDH is a homodimeric enzyme homologous to known dimeric MDHs from other sources. The optimal pH for OAA reduction by MaMDH was pH 8.0 (Figure 4A), which was in accordance with MDH from cyanobacteria *Synechocystis* sp. PCC 6803 (SyMDH) for oxidative reaction (pH 8.0) [22]. This pH optimum is also comparable to that of bacterial MDHs from *E. coli* (pH 8.1), *Bacillus subtilis* (pH 8.0), *Streptomyces aureofaciens* (pH 8.0) and *S. avermitilis* MA-4680 (pH 8.0) [23,24,25], but more alkaline than that of mMDH from fungus *Talaromyces emersonii* (pH 7.5) [26] and cMDHs from the higher plants *Aptenia cordifolia* (pH 7.0) and pineapple (pH 6.8–7.0) [27,28]. Recombinant MaMDH was not highly stable under alkaline conditions, losing nearly 60% of its activity at pH 8.5.

The effect of temperature on enzyme activity is characterized by two principal features: an optimal temperature and a range of thermal tolerance. MaMDH showed maximum activity for oxaloacetate (OAA) reduction at around 40 °C (Figure 4B), which is lower than that of SyMDH (45–50 °C) [22]. This optimum temperature is comparable to that of MDH from *Flavobacterium frigidimaris* (40 °C) [29], but substantially lower than that of bacterial MDHs from *E. coli*, *S. coelicolor* A3(2), *B. subtilis*, *B. stearothermophilus*, and *Salinibacter ruber* (50–67 °C), as well as the thermophilic fungal mitochondrial MDH from *T. emersonii* (52 °C) [23,26,30,31].

Thermal stability of MDHs was generally low, and thermophilic MDHs often exhibit more hydrogen bonds between the protein subunits of polar amino acid residues [32,33]. MDHs from thermophiles, such as *S. coelicolor* A3(2) and *Nitrosomonas europaea,* retained most activity after treatment at 50 °C but were completely inactivated at 60 °C [30,34]. In this study, MaMDH retained approximately 90% and 70% of its maximal activity after incubation at 70 °C and 75 °C, respectively (Figure 4C). Furthermore, thermal inactivation studies showed that MaMDH was thermostable with an estimated half-life (*t*_1/2_) of 220 min at 55 °C (Figure 4D), indicating greater thermostability than most bacterial and many thermophilic fungi MDHs reported to date [35], though less than those from a few extremely thermophile bacteria. For example, MDHs from *Penicillium duponti*, *Sporotrichum thermophile* and *Thermoascus aurantiacus* have half-lives of <10 min at 50 °C; those from *Humicola lanuginose* and *Chaetomium thermophile var. Coprophile* have half-lives of <30 min; and those from *T. emersonii* (*t*_1/2_ = 30 min), *Mucor pusillus* (*t*_1/2_ = 60 min) and *S. coelicolor* A3(2) (*t*_1/2_ = 120 min) show moderate stability [19,26,30,35]. Evidently, MDH from mesophilic cyanobacterium *M. aeruginosa* PCC7806 is a significantly thermostable enzyme.

The primary structure dictates a protein’s higher-order architecture and thermal stability. Hydrophobic interactions drive protein folding, and a compact, ordered hydrophobic core—enriched with larger, branched residues like Leucine (12.6%) and Isoleucine (6.5%) in MaMDH—enhances van der Waals contacts, reduces internal cavities, and improves heat resistance. Conversely, minimizing unstable residues such as Cysteine (1.8% in MaMDH), which can form incorrect disulfides or undergo oxidative degradation, further stabilizes the structure. Additionally, a higher prevalence of charged residues like Arginine (5.5%) and Glutamic acid (5.2%) promotes extensive salt bridge and hydrogen bond networks, contributing to MaMDH’s thermal resilience. Further mechanistic insights will require elucidation of its three-dimensional crystal structure.

Typically, MDHs do not depend on metal ions for normal catalytic function, though their activity can be modulated by metal ion binding. In accordance with this general property, the recombinant MaMDH was characterized as a metal ion-independent enzyme (Table 1). MaMDH was strongly inhibited by Zn^2+^ and Co^2+^, consistent with observations for many fungal, bacterial and plant MDHs, such as mMDH from *Cryptococcus neoformans* [36] and MDH from *S. coelicolor* A3(2), *B. subtilis* and *Phaseolus mungo* [23,30,37], but was completely different from SyMDH, SyMDH was slightly activated by Zn^2+^ and was significantly activated by Co^2+^ [22]. The underlying reasons were unknown and required further investigations. Cu^2+^ inhibited MaMDH, which was similar to SyMDH. MaMDH activity was also repressed by Ni^2+^. Identical to SyMDH, MaMDH activity was promoted in the presence of Mg^2+^, Ca^2+^, Mn^2+^ and Na^+^, respectively. DMSO and Triton X-100 both impaired MaMDH activity at high concentrations in this study (Table 1), with a parallel effect observed for ScMDH [30]. The underlying mechanisms, however, differ: DMSO acts through its sulfinyl group to disrupt hydrophobic domains or secondary structures, while Triton X-100 perturbs protein stability by interfering with hydrophilic surfaces. Despite their different mechanisms, both agents ultimately lead to protein destabilization and functional loss.

While the majority of MDHs employ NADH as a cofactor, the plant chloroplastic isoform utilizes NADPH. Dual-coenzyme specificity has been reported only in archaeon *Metallosphaera sedula* (MsMDH) [6]. MsMDH can utilize both NADH and NADPH as a cofactor, with the *K*_m_^NADH^ and *K*_m_^NADPH^ values of 0.123 and 0.165 mM, respectively, and *k*_cat_ values of 1.201 and 0.614 min^−1^. Compared to MsMDH, the MaMDH reported in this study better fits the characteristics of dual-coenzyme dependency. MaMDH exhibits higher affinity for both coenzymes with *K*_m_^NADH^ and *K*_m_^NADPH^ values of 33.140 and 113.200 μM, respectively, and significantly higher *k*_cat_ values (31.688 s^−1^ and 8.710 s^−1^), making it a more efficient dual-coenzyme-dependent MDH

Phylogenetic studies indicate that plant chloroplast NADP-MDHs are derived from cytosolic NAD^+^-dependent precursors rather than from the respective gene for the plastid NAD^+^-specific isoenzymes, and cyanobacterial MDHs representing the most ancient branch of the MDH family [7]. This evolutionary path aligns with current understanding of endosymbiotic gene transfer events between ancient photosynthetic organisms and plant ancestors. Based on our experimental findings and the endosymbiosis origin theory, we propose that the ancestral precursor of chloroplast NADP-MDH originated from an ancient cyanobacterial dual-coenzyme-dependent MDH, and the emergence of NADP^+^-dependency is an adaptive evolutionary phenotype co-evolving with chloroplast organellogenesis and functional specialization. According to the endosymbiotic origin hypothesis, chloroplast evolved from ancient cyanobacteria that were engulfed by the primitive archaeplastidal cells and subsequently underwent gradual functional specialization. During this organelle evolutionary process, the ancestral dual-coenzyme MDH of ancient cyanobacteria lost its NAD^+^-dependent activity—presumably because it was no longer required to participate in NAD^+^-based energy metabolism—while specializing to exclusively provide reducing power (via NADP^+^) for photosynthetic anabolism. Consequently, the enzyme progressively lost its dependence on NAD^+^ while retaining and enhancing its specificity for NADP^+^, ultimately evolving into the modern chloroplast NADP-MDH. This shift in coenzyme specificity could have been achieved through a limited number of key amino acid substitutions within the Rossmann-fold cofactor-binding domain of MDH.

Owing to its central role in metabolism, the evolution of this core enzyme was likely a stepwise process, conferring new functionalities gradually and leaving traceable evolutionary signatures [16]. The dual-coenzyme dependence of MaMDH may represent an evolutionary intermediate left during the process of MDH enzymes evolution. One method to validate an evolutionary pathway is reverse engineering. In this study, the construction of four mutants (T3, T4, and T7) revealed that a limited number of amino acid substitutions within the coenzyme-binding site were sufficient to alter the cofactor specificity of MaMDH from a predominant affinity for NAD^+^ to a strongly enhanced preference for NADP^+^. These functional shifts provide compelling evidence supporting the hypothesized evolutionary mechanism governing MDH coenzyme specificity.

Currently, the high-resolution three-dimensional structure of MDH from cyanobacterium remains undetermined. In this study, using bioinformatic analysis—including protein secondary structure prediction and sequence alignment—we proposed that the primary cofactor specificity determinant motif in MaMDH is Asp^44^-Ile-Val-His-Gly-Leu-Pro^50^. Using site-directed mutagenesis, we replaced this amino acid sequence with the corresponding NADP^+^-preferring motif typically found in NADP^+^-dependent MDHs. This substitution successfully altered the cofactor specificity of MaMDH, confirming the hypothesized role of this region in cofactor binding. Similar cofactor specificity switches have been reported for MDHs from *T. flavus* (TfMDH) and *S. coelicolor* (ScMDH) [20,21,30]. However, unlike the complete reversal of cofactor preference from NAD^+^ to NADP^+^ observed in TfMDH and ScMDH, only partial conversion was achieved for MaMDH in our experiments.

Previous studies have established that a highly conserved residue at the C-terminus of the β2-strand within the cofactor specificity domain (β2-α2-β3) of the Rossmann fold is a critical determinant of cofactor specificity in the dehydrogenase family [38,39]. NAD^+^-dependent dehydrogenases generally possess a conserved acidic residue (Asp or Glu) at this position, while NADP^+^-dependent enzymes typically have a neutral glycine (Gly) [5,21,38]. Secondary structure alignment indicated that MaMDH contains an Asp residue at the C-terminus of its β2-strand within the cofactor-binding domain, consistent with the canonical NAD^+^-dependent dehydrogenases. However, our study demonstrated that MaMDH retains measurable catalytic activity with NADPH as a cofactor. This finding implies the existence of additional, yet unidentified, residue(s) within MaMDH that contribute specifically to NADP^+^ recognition and binding. Confirming the identity and role of these putative NADP^+^-binding site(s) will require further structural investigation via protein crystallography.

## 4. Materials and Methods

### 4.1. Protein Expression and Purification

The coding sequence of MaMDH was amplified by polymerase chain reaction (PCR) and ligated into the pET-28b expression vector. The resulting recombinant plasmid, pET-28b-MaMDH, was transformed into *Escherichia coli* Rosetta (DE3) cells. Transformed colonies were cultivated in Luria-Bertani (LB) broth supplemented with kanamycin (30 μg/mL) and chloramphenicol (30 μg/mL) at 37 °C until the OD_600_ reached 0.4–0.6. Protein expression was induced with 0.5 mM isopropyl β-d-1-thiogalactopyranoside (IPTG) at 20 °C for 20 h. Cells were then harvested by centrifugation at 5000× *g* for 20 min at 4 °C. The pellet was resuspended in ice-cold LEW buffer (50 mM NaH_2_PO_4_, 300 mM NaCl, pH 8.0) and lysed by ultrasonication on ice. Following centrifugation at 12,000× *g* for 20 min at 4 °C, the soluble supernatant was applied to a Co^2+^-affinity chromatography column (Cat. #635502, Clontech, TaKaRa, Dalian, China) for purification of the His_6_-tagged MaMDH protein. All purification steps were carried out at 4 °C. Protein concentration was determined using a Bio-Rad protein assay kit (Bio-Rad, Hercules, CA, USA).

### 4.2. Molecular Mass Determination

To determine the subunit molecular mass, the purified protein was resolved by SDS–PAGE under denaturing conditions using a discontinuous system with a 5% stacking gel and a 12% separating gel. The preliminary molecular mass of active MaMDH was determined by gel filtration chromatography on a Superdex 200 10/300 Increase column (GE Healthcare Life Sciences, Pittsburgh, PA, USA). The column was equilibrated with 50 mM potassium phosphate buffer (pH 7.0) containing 150 mM NaCl and 0.01% sodium azide. A calibration curve was generated using the following protein standards: ovalbumin (44 kDa), conalbumin (75 kDa), aldolase (158 kDa), ferritin (440 kDa), and thyroglobulin (669 kDa). The apparent molecular mass of the enzyme was determined by matrix-assisted laser desorption/ionization–time-of-flight mass spectrometry (MALDI-TOF-MS) on a 5800 Analyzer (AB SCIEX, Old Connecticut Path Framingham, MA, USA), following a previously described method [40]. Briefly, 0.6 µL of sample was spotted directly onto the target plate, mixed with matrix solution (1:3, *v*/*v*), and allowed to dry at ambient temperature before analysis. Mass spectra were acquired and processed using the TOF-TOF Series Explorer™ software (version 4.0, AB SCIEX).

### 4.3. Enzyme Kinetic Assay

The enzyme activity was routinely assayed in a 1 mL reaction volume at 25 °C and pH 8.0 by monitoring the NAD(P)H change at 340 nm wavelength with a thermostat, MAPADA P7 UV-visible spectrophotometer (MAPADA, Shanghai, China), using a molar extinction coefficient of 6.22 mM^−1^·cm^−1^. Standard reaction mixtures contained 100 mM Tris-HCl (pH 8.0), 0.25 mM OAA, 0.125 mM NAD(P)H. One unit (U) of enzyme activity was defined as the generation of 1 µM NAD(P)^+^ per min. The Michaelis constant (*K*_m_) for NAD(P)^+^ was determined by measuring initial rates at varying concentrations of oxaloacetate (OAA). Apparent kinetic parameters were derived through nonlinear regression analysis performed with Prism 7.0 software (GraphPad Software, San Diego, CA, USA). All assays were carried out in three independent replicates.

### 4.4. Enzyme Characterization

The pH-dependent activity of recombinant MaMDH was assayed in 100 mM Tris-HCl buffer (pH 7.5–9.5) at 25 °C. To evaluate the effect of temperature on enzyme activity, assays were conducted between 25 °C and 55 °C at pH 8.0. Thermal stability was assessed by incubating enzyme aliquots at temperatures ranging from 25 °C to 80 °C (pH 8.0) for 20 min, followed by rapid cooling on ice and measurement of residual activity under standard assay conditions. Additionally, thermostability at 55 °C was specifically examined. The influence of metal ions (K^+^, Na^+^, Li^+^, Rb^+^, Zn^2+^, Mn^2+^, Mg^2+^, Ca^2+^, Co^2+^, Cu^2+^, and Ni^2+^; each at 2 mM) and organic solvents (dimethyl sulfoxide and Triton X-100; each at various vol/vol concentrations) on MaMDH activity was determined by incorporating the respective compounds into the reaction mixture. All activity measurements were performed in at least three independent replicates.

### 4.5. Site-Directed Mutagenesis and Circular Dichroism Spectroscopy

Site-directed mutagenesis was performed using the QuikChange kit (Stratagene, La Jolla, CA, USA). Mutant proteins were purified following the same procedure as the wild-type, and their enzymatic activity was assessed under identical conditions with 0.3 mM NADH. Circular dichroism (CD) spectra were recorded on a Jasco J-810 spectropolarimeter (JASCO Shanghai, Shanghai, China). Samples of MaMDH and its mutants were diluted to 0.2 mg/mL in a CD buffer consisting of 20 mM NaH_2_PO_4_ and 75 mM Na_2_SO_4_ (pH 7.5). A volume of 200 µL of each protein sample was loaded into a microcuvette and scanned from 190 to 280 nm. The mean residue ellipticity ([θ], in deg·cm^2^·dmol^−1^) was calculated using the equation: [θ] = θ/[10 · (*n* − 1) · C · l], where θ represents the measured ellipticity (in mdeg), *n* is the number of amino acid residues, C denotes the molar concentration, and l is the path length (0.1 cm). For each sample, three scans were averaged.

## 5. Conclusions

The present study provides, for the first time, evidence that cyanobacterial MaMDH exhibits dual-coenzyme dependency. Based on our experimental findings, we propose that the ancestral NADP^+^-MDH originated from ancient cyanobacterial dual-coenzyme-dependent MDH, and NADP^+^ dependency in the MDH family is an adaptation phenotype co-evolving with chloroplast specialization. In this study, four mutants were constructed based on the speculated cofactor specificity determinant motif in MaMDH, and only a few site-directed mutations were required to achieve modification of the coenzyme specificity for MaMDH, which indicates that our speculation is reasonable. Moreover, we conducted a detailed biochemical and enzymatic property analysis of MaMDH. MaMDH was found to be a homodimer enzyme with considerable thermal stability. This current work provides new insights into the molecular evolution mechanisms of coenzyme specificity within the MDH family.

## Figures and Tables

**Table 1:** Effect of different metal ions on the activity of MaMDH.

Metal Ions	Relative Activity (%)	Metal Ions and Compounds	Relative Activity (%)
None	100.00 ± 0.00	Co^2+^	8.73 ± 0.36
Na^+^	106.23 ± 1.95	Cu^2+^	79.07 ± 1.62
K^+^	99.52 ± 0.46	Ca^2+^	130.77 ± 1.99
Li^+^	106.95 ± 1.99	DMSO (2%)	97.13 ± 0.54
Rb^+^	115.26 ± 3.22	DMSO (4%)	85.75 ± 0.82
Zn^2+^	0.00 ± 0.00	DMSO (8%)	69.53 ± 1.93
Mg^2+^	135.84 ± 0.35	Triton X-100 (2%)	85.00 ± 0.75
Mn^2+^	128.14 ± 5.12	Triton X-100 (4%)	69.31 ± 1.97
Ni^2+^	14.94 ± 0.49	Triton X-100 (8%)	50.06 ± 1.19

**Table 2:** The kinetic parameters of the wild-type MaMDH and its mutants.

Enzyme	NADH			NADPH			Specificity		Degree of Alteration
	*K*_m_	*k*_cat_	*k*_cat_/*K*_m_ (A)	*K*_m_	*k*_cat_	*k*_cat_/*K*_m_ (B)	A/B	B/A	
	(μM)	(s^−1^)	(μM^−1^·s^−1^)	(μM)	(s^−1^)	(μM^−1^·s^−1^)			
MaMDH-WT	33.140 ± 0.95	31.688 ± 1.43	0.956	113.200 ± 3.46	8.710 ± 1.45	0.077	12.427	0.080	1.00
MaMDH-T_1_ D^44^G	26.160 ± 2.17	22.361 ± 1.75	0.855	147.700 ± 12.04	15.642 ± 1.73	0.106	8.071	0.124	1.54
MaMDH-T_3_ D^44^G/I^45^S/G^48^S	179.600 ± 11.60	22.498 ± 2.37	0.125	38.230 ± 1.58	24.283 ± 1.20	0.635	0.197	5.071	63.01
MaMDH-T_4_ D^44^G/I^45^S/H^47^R/G^48^S	177.000 ± 4.83	27.131 ± 1.16	0.153	25.880 ± 1.13	26.542 ± 1.04	1.026	0.149	6.691	83.15
MaMDH-T_7_ D^44^G/I^45^S/V^46^E/H^47^R/G^48^S/L^49^F/P^50^Q	136.400 ± 6.31	46.095 ± 3.06	0.338	43.110 ± 3.22	54.110 ± 2.31	1.255	0.269	3.714	46.16

## Data Availability

The original contributions presented in the study are included in the article. Further inquiries can be directed to the corresponding author.
